# Presence of *Cryptosporidium parvum* and *Giardia lamblia* in water samples from Southeast Asia: towards an integrated water detection system

**DOI:** 10.1186/s40249-016-0095-z

**Published:** 2016-01-13

**Authors:** Thulasi Kumar, Mohamad Azlan Abd Majid, Subashini Onichandran, Narong Jaturas, Hemah Andiappan, Cristina C. Salibay, Hazel A. L. Tabo, Norbel Tabo, Julieta Z. Dungca, Jitbanjong Tangpong, Sucheep Phiriyasamith, Boonyaorn Yuttayong, Raxsina Polseela, Binh Nhu Do, Nongyao Sawangjaroen, Tian-Chye Tan, Yvonne A. L. Lim, Veeranoot Nissapatorn

**Affiliations:** Department of Parasitology (Southeast Asia Water Team), Faculty of Medicine, University of Malaya, Kuala Lumpur, Malaysia; Biological Science Department, College of Science and Computer Studies, De La Salle University-Dasmariñas, Dasmariñas, Philippines; School of Science and Technology, Centro Escolar University, Manila, Philippines; School of Allied Health Sciences and Public Health, Walailak University, Nakhon Si Thammarat, Thailand; Graduate School, Kasem Bundit University, Bangkok, Thailand; Regional Medical Sciences Center, Department of Medical Sciences, Ministry of Public Health, Nakhon Ratchasima, Thailand; Department of Microbiology and Parasitology, Faculty of Medical Science, Naresuan University, Phitsanulok, Thailand; Department of Parasitology, Faculty of Medicine, Vietnam Military Medical University, 160 Phung Hung Road, Phuc La Ward, Ha Dong District, Hanoi Vietnam; Department of Microbiology, Faculty of Science, Prince of Songkla University, Hat Yai, Thailand

**Keywords:** *Cryptosporidium parvum*, *Giardia lamblia*, Physicochemical, Microscopy, Real-time polymerase chain reaction, Southeast Asia

## Abstract

**Background:**

Access to clean and safe drinking water that is free from pathogenic protozoan parasites, especially C*ryptosporidium parvum* and *Giardia lamblia* that cause gastrointestinal illness in humans, is still an issue in Southeast Asia (SEA). This study is the first attempt to detect the aforementioned protozoan parasites in water samples from countries in SEA, using real-time polymerase chain reaction (qPCR) assays.

**Methods:**

A total of 221 water samples of 10 l each were collected between April and October 2013 from Malaysia (53), Thailand (120), the Philippines (33), and Vietnam (15). A physicochemical analysis was conducted. The water samples were processed in accordance with the US Environmental Protection Agency’s methods 1622/1623.1, microscopically observed and subsequently screened using qPCR assays.

**Results:**

*Cryptosporidium* oocysts were detected in treated water samples from the Philippines (1/10), with a concentration of 0.06 ± 0.19 oocyst/L, and untreated water samples from Thailand (25/93), Malaysia (17/44), and the Philippines (11/23), with concentrations ranging from 0.13 ± 0.18 to 0.57 ± 1.41 oocyst/L. *Giardia* cysts were found in treated water samples from the Philippines (1/10), with a concentration of 0.02 ± 0.06 cyst/L, and in untreated water samples from Thailand (20/93), Vietnam (5/10), Malaysia (22/44), and the Philippines (16/23), with concentrations ranging from 0.12 ± 0.3 to 8.90 ± 19.65 cyst/L. The pathogens *C. parvum* and *G. lamblia* were detected using using qPCR assays by targeting the 138-bp fragment and the small subunit gene, respectively. *C. parvum* was detected in untreated water samples from the Philippines (1/23) and Malaysia (2/44), whilst, *G. lamblia* detected was detected in treated water samples from the Philippines (1/10) and in untreated water samples from Thailand (21/93), Malaysia (12/44), and the Philippines (17/23). Nitrate concentration was found to have a high positive correlation with (oo)cyst (0.993).

**Conclusion:**

The presence of (oo)cysts in the water samples means that there is potential risk for zoonotic disease transmission in the studied countries. Detection using qPCR is feasible for quantifying both pathogenic *C. parvum* and *G. lamblia* in large water samples.

## Background

*Cryptosporidium parvum (C. parvum)* and *Giardia lamblia (G. lamblia)* are protozoan parasites that can cause gastrointestinal illness in humans [1]. Both parasites can be transmitted through water in environments where there are poor sanitation systems, lack of hygiene, an inadequate water management system, and wastewater reuse practices. In recent decades, waterborne outbreaks of cryptosporidiosis and giardiasis have been the most prevalent infections reported in countries such as North America, England, Scotland, and Australia [2]. The existence of protozoans in open water reservoirs and treated water supply is mainly due to the contamination of the environmentally resistant of *Cryptosporidium* oocyst and *Giardia* cyst stages. However, the aforementioned condition is highly unaffected in harsh water conditions or disrupted by conventional water disinfection treatments (i.e. chlorination, filtration, etc.) due to its resistance [3].

All over the world, *Cryptosporidium* spp. and *Giardia* spp. are two of the highest reported causative parasitic waterborne agents [4], but these two protozoans are especially common waterborne outbreaks in the USA. Cryptosporidiosis and giardiasis can be primarily transmitted via direct contact with contaminated water (diving, swimming, bathing, etc.), [5] via contact with water that has been deficiently treated [6], and via accidental ingestion of water containing (oo)cysts [7]; infection with either can lead to potentially fatal diseases in humans.

The immunomagnetic separation (IMS) technique, which was developed by The United States Environmental Protection Agency (EPA) can be used to morphologically identify both protozoan parasites. However, identification at species-level can only be done using molecular techniques, such as those used in polymerase chain reaction (PCR)-restriction fragment length polymorphism [8, 9] and nested PCR [10, 11]. These techniques can help to determine the prevalence and contamination level of certain protozoan species, and having this information can then help policymakers put in place preventive measures to eradicate the spread or proliferation of pathogenic species [12]. Generally, untreated water is more likely to be contaminated with protozoan parasites due to poor sanitation, but treated water can also be vulnerable to (oo)cyst contamination, as a result of an inefficient water management system.

This study is the first attempt to detect the aforementioned protozoan parasites in water samples from countries in Southeast Asia (SEA), using real-time PCR (qPCR) assays. Since it was developed in 1992 by Higuchi and coworkers [13], qPCR assay has widely been used for diagnosis in laboratories, and has replaced conventional PCR, which is less sensitive in distinguishing between different species due to slight differences occurring at the nucleotide level. Real-time PCR can detect parasites in large samples (without onsite filtration) over a shorter time period (three hours) [11, 14]. Previous studies have indicated the successful detection of both parasites using qPCR assays in different assays in different types of water samples (i.e. sewage, swimming pools) [15–17]. The minimum detection limit reported for *G. lamblia* and *C. parvum* was two cysts and one oocyst, respectively, in 20–1,500 l of spiked water samples [18].

Because the data on water contamination with protozoan parasites is limited, this study aims to examine the current distribution of waterborne protozoan parasites in various types of water samples from four countries in SEA, namely Malaysia, Thailand, the Philippines, and Vietnam. The distribution patterns of *C. parvum* and *G. lamblia* in the water basins of these countries would indicate if there is potential risk for zoonotic disease transmission in the region.

## Methods

### Study site and sampling procedure

**Fig. 1:**
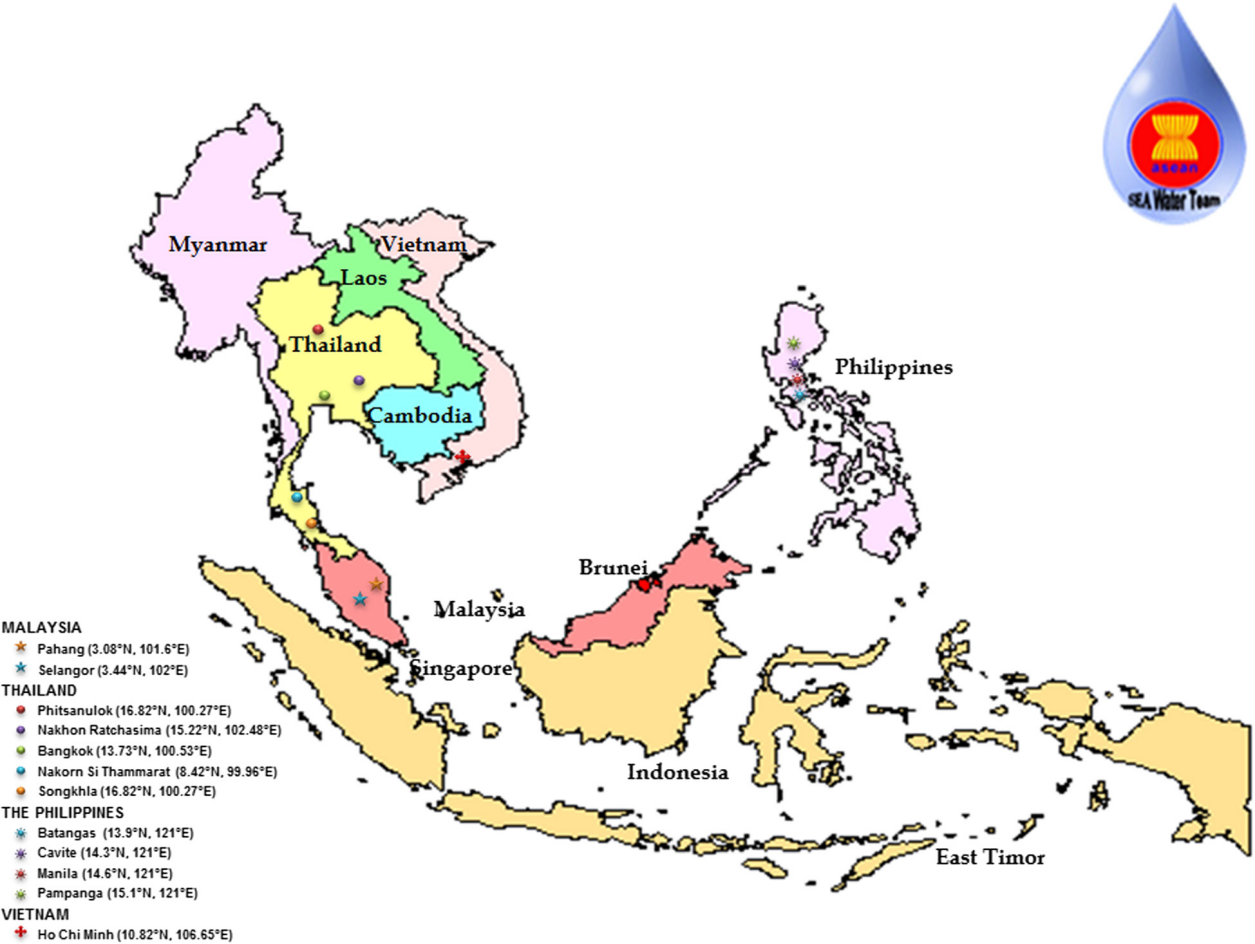
Sampling locations in Malaysia, Thailand, and Vietnam used in this study, together with previously studied samples from Malaysia (Selangor), Thailand (Songkhla), and the Philippines, used for molecular analysis

**Fig. 2:**
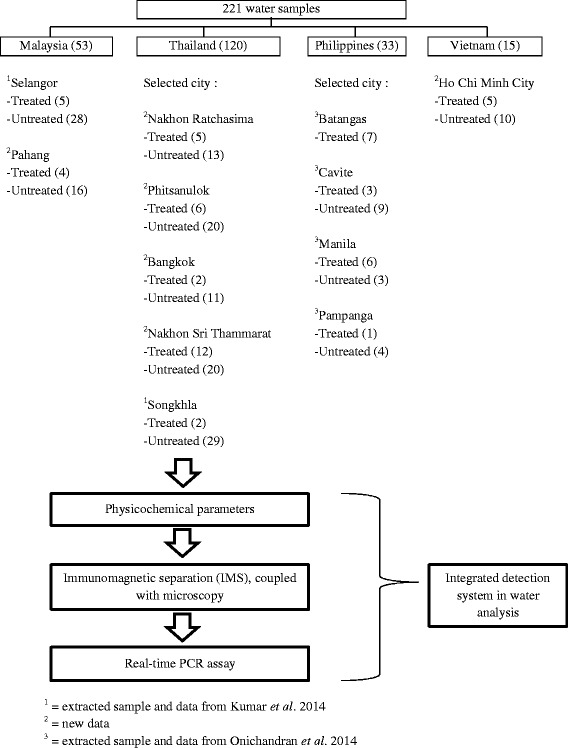
Flowchart showing the overall analysis of the water samples

A total of 221 water samples were collected between April and October 2013; 53 samples were collected from Malaysia, 120 samples from Thailand, 33 samples from the Philippines, and 15 samples from Vietnam. They comprised both treated and untreated water (see Figs. 1 and 2).

Treated water samples included any drinking water processed via demineralization, reverse osmosis, ozonizations, and UV radiation (i.e. drinking, dispensed, and mineral), as well tap water, which is supplied to buildings after treatment via coagulation, flocculation, sedimentation, filtration, and disinfection. Swimming pool water was also included in the treated water category because pools undergo a treatment process that includes purification, flocculation, filtration, and chemical disinfection (i.e. chlorine, sodium hypochlorite, copper silver ionization, or hydrogen peroxide). In the untreated water category, water samples were collected from rain, springs, wells, recreational water bodies (i.e. lakes, waterfalls, rivers and streams that branch off rivers, such as canals or channels), and effluent water sources.

### Physicochemical analysis of the water samples

Physical parameters (i.e. turbidity, total dissolved solids [TDS], salinity, and dissolved oxygen [DO]) were initially measured in situ using a multiprobe parameter (YSI 556 Multiprobe System, Ohio, USA) at the sampling sites. Meanwhile, chemical parameters (i.e. ammonia, chlorine, nitrate, and nitrite) were measured using a colorimeter (DR/890 Portable Colorimeter, Colorado, USA). Both physical and chemical (physicochemical) parameters were recorded as mean values.

### Sample concentration and purification

Each of water was transported to a laboratory within 24 h of collection and filtered through a flatbed membrane filtration machine (EMD Millipore Corp., Billerica, Massachusettes, USA), using a 142 mm diameter and a 1.2 μm pore-sized nitrocellulose membrane (EMD Millipore Corp., Massachusetts, USA). The accumulated sediments were removed from the top of the membrane using a 0.1 % Tween 80 solution and collected in a Falcon™ 15 ml tube (Brookings, South Dakota, USA). The 10 ml eluate was then subjected to the IMS procedure.

### Immunomagnetic separation (IMS) and microscopic examination

The EPA-established method 1623.1 [19] was employed to detect *Cryptosporidium* and *Giardia* (oo)cysts. The eluate was purified using the IMS technique (Dynabeads® GC-Combo, Carlsbad, USA). Magnetic beads with *Cryptosporidium* and *Giardia*-specific monoclonal antibodies were magnetized, separating the (oo)cyst-magnetic beads complex from the debris [20]. Finally, 50 μL of the final product was separated into two portions: one for microscopic examination and the other for molecular analysis.

Each microscopy well slide (Invitrogen Dynal AS, Oslo, Norway) was prepared with 5 μl of 1 N NaOH solution, and then 25 μL of the sample was spotted onto a well. The slide was then stained with fluorescein isothiocyanate-conjugated (FITC) *Giardia/Cryptosporidium* monoclonal antibody reagent (Cellabs, New South Wales, Australia) and 4′, 6′-diamidino-2-phenylindole (DAPI) solution (Sigma-Aldrich, St. Louis, MO, USA), before being fixed with methanol. The slides were then examined through an epifluorescence microscope (Olympus Model BX-51, Tokyo, Japan), alongside positive (Cellabs, New South Wales, Australia) and negative (purified water) controls. Morphology was determined based on round oocysts (4–6 μm) for *Cryptosporidium* and oval cysts (9–12 μm) for *Giardia*. The numbers of *Giardia* cysts and *Cryptosporidium* oocysts were calculated as follows:

$$ \mathrm{number}\ \mathrm{o}\mathrm{f}\ \left(\mathrm{o}\mathrm{o}\right)\mathrm{cysts}\ \mathrm{per}\ \mathrm{liter}=\mathrm{number}\ \mathrm{o}\mathrm{f}\ \left(\mathrm{o}\mathrm{o}\right)\mathrm{cysts}\ \mathrm{o}\mathrm{n}\ \mathrm{slide}\ \left(\mathrm{contained}\ \mathrm{b}\mathrm{y}\ 25\ \upmu \mathrm{L}\ \mathrm{after}\ \mathrm{I}\mathrm{M}\mathrm{S}\ \mathrm{concentration}\right)/10\ \mathrm{L}\ \mathrm{o}\mathrm{f}\ \mathrm{collected}\ \mathrm{sample}. $$

### DNA extraction, purification, and concentration

The end product of the remaining samples were frozen in liquid nitrogen and thawed in a hot water bath at 56 °C five times before overnight incubation for 12 h to break the tough (oo)cyst cell wall. Then, they were subjected to the standard DNA extraction method (QIAamp DNA Mini Kit, Qiagen Inc., Hilden, Germany). All extracted DNA were measured for purity and concentration using a fluorescence spectrophotometer (Micro UV–vis., ES-2 model; Malcom, Tokyo, Japan). The DNA that did not fall within the purity range of 1.7–2.2 were re-concentrated and purified following the protocol for DNA cleanup using Genomic-tips (QIAamp DNA Mini Kit, Qiagen Inc., Hilden, Germany).

### Real-time polymerase chain reaction (qPCR)

The primers and probe sequences for *C. parvum* (AF188110; CrF: 5′-CGC TTC TCT AGC CTT TCA TGA-3′, CrR: 5′-CTT CAC GTG TGT TTG CCA AT-3′, *Cryptosporidium*: Texas Red-5′ CCA ATC ACA GAA TCA TCA GAA TCG ACT GGT ATC 3′-BHQ2) were used to amplify a 138-bp fragment inside the *C. parvum* specific 452-bp. For *G. lamblia*, the primers and probe sequences (M54878; *Giardia*-80 F: 5′-GAC GGC TCA GGA CAA CGG TT-3′, *Giardia*-127R: 5′-TTG CCA GCG GTG TCC G-3′, and *Giardia* 150 T: FAM-5′ CCC GCG GCG GTC CCT GCT AG 3′-BHQ1) were chosen to specifically detect a 62-bp fragment within the SSU RNA gene [21, 22].

Singleplex qPCR assays were amplified in a concentration with a volume of 25 μL, consisting of: PCR premix of 2 x SensiFAST™ Probe No-ROX Kit (Bioline Ltd, London, United Kingdom), 3.75-pmol of each primer, 1.25-pmol of probe, and 2.5-μL of DNA template. Amplification was performed using the CFX96™ system (Bio-Rad, California, USA), and consisted of five minutes at 95 °C, followed by 45 cycles of 10 s at 95 °C, 20 s at 55 °C, and 20 s at 72 °C. Fluorescence was measured during the annealing step of each cycle.

### Quality control for qPCR

The sample’s first signal threshold cycle (Ct) value was quantified by comparing it with the standard reference Ct value of the positive control. The positive control either originated from the *G. lamblia* axenic culture broth sample (courtesy of Dr. Nongyao Sowangjaroen, Department of Microbiology, Prince of Songkla University, Thailand), or from the seeded *Cryptosporidium* oocyst samples that were prepared following the EasySeed™ *Cryptosporidium* only procedure (TCS Bioscience Ltd, Buckingham, United Kingdom). At the threshold peak, fluorescence emitted at 490 nm for *G. lamblia* and at 575 nm for *C. parvum*.

## Results

### Baseline data from the physicochemical analysis

Physicochemical data for both treated and untreated water samples were computed as mean ± standard deviation (SD), with 95 % confidence intervals (CIs), as shown in Table 1.

**Table 1:** The physicochemical parameter(s) of water samples in selected SEA countries

Country	Type of water	Average, SD, and CI (95%) values	Water quality parameters (physicochemical)							
			Physical				Chemical			
			Turbidity	TDS	Salinity	DO	Chlorine	Nitrate	Nitrite	Ammonia
			(NTU)	(mg/L)	(ppt)	(mg/L)	(mg/L)	(mg/L)	(mg/L)	(mg/L)
Malaysia	Treated	Average	3.04	65.90	0.05	3.01	0.18	0.14	0.08	2.07
		SD	5.05	88.65	0.05	3.30	0.35	0.19	0.15	5.63
		CI (95%)	3.50	61.43	0.03	2.28	0.24	0.13	0.11	3.90
	Untreated	Average	17.79	58.78	0.05	2.56	0.15	0.32	0.20	0.37
		SD	23.34	66.48	0.04	2.15	0.16	0.41	0.38	0.51
		CI (95%)	7.96	22.68	0.01	0.73	0.05	0.14	0.13	0.17
Thailand	Treated	Average	1.34	416.53	0.30	5.24	0.24	2.31	0.03	0.69
		SD	1.98	1170.86	0.92	6.18	0.76	8.03	0.04	2.90
		CI (95%)	0.85	500.77	0.39	2.64	0.32	3.43	0.02	1.24
	Untreated	Average	62.51	131.30	1.39	3.00	0.24	0.29	0.23	0.17
		SD	149.91	198.24	4.98	1.88	0.30	0.39	1.41	0.23
		CI (95%)	29.53	39.05	0.98	0.37	0.06	0.08	0.28	0.05
Philippines	Treated	Average	1.04	306.13	0.23	0.84	0.58	0.10	0.01	0.12
		SD	1.08	254.15	0.19	0.93	0.74	0.20	0.01	0.17
		CI (95%)	0.75	176.11	0.13	0.65	0.52	0.14	0.00	0.11
	Untreated	Average	8.84	530.39	0.54	0.64	0.28	0.18	0.20	0.43
		SD	6.38	902.59	1.00	2.28	0.50	0.25	0.32	0.32
		CI (95%)	2.61	368.87	0.41	0.93	0.20	0.10	0.13	0.13
Vietnam	Treated	Average	0.31	224.92	0.06	4.82	0.04	0.09	0.01	0.10
		SD	0.38	376.10	0.06	0.98	0.06	0.05	0.01	0.01
		CI (95%)	0.37	368.58	0.06	0.96	0.06	0.05	0.01	0.01
	Untreated	Average	23.60	3630.54	2.77	3.83	0.21	0.56	0.58	0.34
		SD	21.86	3546.22	3.19	2.28	0.17	0.42	0.39	0.29
		CI (95%)	13.55	2197.93	1.98	1.41	0.11	0.26	0.24	0.18

Treated water includes drinking water, water dispenser, mineral water, tap water and swimming pools

Untreated water includes rain water, springs, wells, recreational lake, rivers, waterfalls, canals/channels and effluent water

The highest ammonia reading was recorded in treated water samples from Malaysia, 2.07 ± 5.63 mg/L (CI: 3.90), whereas the highest reading of chlorine was recorded in treated water samples from the Philippines, 0.58 ± 0.74 mg/L (CI: 0.52). Two treated water samples from Thailand showed maximum levels of DO and nitrate, at 5.24 ± 6.18 mg/L (CI: 2.64) and 2.31 ± 8.03 mg/L (CI: 3.43), respectively, whereas untreated water samples from Thailand showed the highest average level of turbidity, at 62.51 ± 149.91 NTU (CI: 29.53). Untreated water samples from Vietnam showed maximum levels of TDS (3630.54 ± 3546.22 mg/L; CI: 2197.93) and salinity (2.77 ± 3.19 ppt, CI: 1.98), and treated water samples showed maximum levels of nitrite (0.58 ± 0.39 mg/L; CI: 0.24).

### Detection of *Cryptosporidium* and *Giardia*

#### IMS/microscopic identification of *Cryptosporidium* and *Giardia* (oo)cysts

The staining protocol used is based on the concept of conjugated monoclonal antibody with specific FITC dye by targeting the protein of the (oo)cyst cell wall. Meanwhile, DAPI permits the observation of the internal structure of both sporozoites and trophozoites, indicating the viability of individual (oo)cysts [20].

Table 2 shows that *Cryptosporidium* oocysts were detected in both treated and untreated water samples, from the Philippines (57.8 %; 10 % for treated and 47.8 % for untreated water sample); Malaysia (38.6 %; 0 % for treated and 38.6 % for untreated water sample); and Thailand (26.9 %; 0 % for treated and 26.9 % for untreated water sample). Meanwhile, *Giardia* cysts were detected in both treated and untreated samples from all four countries: the Philippines (79.6 %; 10 % for treated and 69.6 % for untreated water sample); Malaysia (50 %; 0 % for treated and 50 % for untreated water sample); Vietnam (50 %; 0 % for treated and 50 % for untreated water sample); and Thailand (21.5 %; 0 % for treated and 21.5 % for untreated water sample), as shown in Table 2.

**Table 2:** The prevalence of *Cryptosporidium* and *Giardia* (oo)cyst under microscopic examination with IMS technique in selected SEA countries

Country	Type of water	No. of samples	*Cryptosporidium*		*Giardia*	
			Positive sample n(%)	Mean concentration (oocyst/L)	Positive sample n(%)	Mean concentration (cyst/L)
Malaysia	Treated	9	N/D^a^	N/D	N/D	N/D
	Untreated	44	17 (38.6)	0.57 ± 1.41	22 (50.0)	0.92 ± 1.74
Thailand	Treated	27	N/D	N/D	N/D	N/D
	Untreated	93	25 (26.9)	0.22 ± 0.59	20 (21.5)	0.12 ± 0.3
Philippines	Treated	10	1 (10.0)	0.06 ± 0.19	1 (10.0)	0.02 ± 0.06
	Untreated	23	11 (47.8)	0.13 ± 0.18	16 (69.6)	8.90 ± 19.65
Vietnam	Treated	5	N/D	N/D	N/D	N/D
	Untreated	10	N/D	N/D	5 (50.0)	0.51 ± 0.81

Treated water includes drinking water, water dispenser, mineral water, tap water and swimming pools

Untreated water includes rain water, springs, wells, recreational lake, rivers, waterfalls, canals/channels and effluent water

^a^N/D means not detected

#### Real-time PCR

The number of cycles required to intersect the threshold level during amplification, which reflects the accumulation of desired fluorescent signals for both *C. parvum* and *G. lamblia*, were computed as Ct values. Coefficient of variation (CV) was used to express consistency between cycles as follows:

$$ \mathrm{Formula}\ \mathrm{f}\mathrm{o}\mathrm{r}\ \mathrm{C}\mathrm{V}\ \mathrm{o}\mathrm{f}\ \mathrm{C}\mathrm{t}\ \mathrm{value}=\mathrm{Standard}\ \mathrm{deviation}\ \mathrm{o}\mathrm{f}\ \mathrm{C}\mathrm{t}/\mathrm{Mean}\ \mathrm{o}\mathrm{f}\ \mathrm{C}\mathrm{t} $$

The optimum Ct value for *C. parvum* was 26.40, compared to a value of 16.26 on the second consecutive amplification cycle and 38.54 on the tenth consecutive amplification cycle. Similarly, for *G. lamblia*, the optimum Ct value was 28.99, compared to a value of 20.15 on the second amplification cycle and 38.47 on the tenth cycle. The mean values of the 10 consecutive runs were 27.63 ± 7.63 for *C. parvum* and 29.14 ± 6.09 for *G. lamblia*, as shown in Table 3.

**Table 3:** Ct values of *C. parvum* and *G. lamblia* after 10 serial dilution in real-time PCR

Serial dilution	Ct value	
	*C. parvum*	*G. lamblia*
10^7^	16.26	20.15
10^6^	19.18	22.21
10^5^	21.55	24.04
10^4^	23.43	27.5
Positive control	26.4	28.99
10^2^	28.89	29.07
10^3^	30.55	30.25
10^8^	35.21	34.33
10^9^	36.57	36.86
10^10^	38.54	38.47

### Detection of *Cryptosporidium* spp.

#### Treated water

*Cryptosporidium* oocysts were detected only in treated water samples from the Philippines; 10 % of the samples tested positive with a concentration of 0.06 ± 0.19 oocyst/L, as shown in Table 2. None of the positive samples detected using the IMS technique tested positive for *C. parvum* using the qPCR method, as shown in Table 4.

**Table 4:** Ct values observed for real-time PCR assay in selected SEA countries

Country	Type of water	No. of samples	*C. parvum*			*G. lamblia*		
			Positive sample n(%)	Mean Ct value	CV	Positive sample n(%)	Mean Ct value	CV
Malaysia	Treated	9	N/D^a^	N/D	N/D	N/D	N/D	N/D
	Untreated	44	2 (4.5)	23.97 ± 3.8	0.16	12 (27.3)	30.49 ± 5.4	0.18
Thailand	Treated	27	N/D	N/D	N/D	N/D	N/D	N/D
	Untreated	93	N/D	N/D	N/D	21 (22.6)	32.03 ± 4.7	0.15
Philippines	Treated	10	N/D	N/D	N/D	1 (10.0)	32.26 ± 0	0
	Untreated	23	1 (4.3)	36.53 ± 0	0	17 (73.9)	34.41 ± 3.5	0.10
Vietnam	Treated	5	N/D	N/D	N/D	N/D	N/D	N/D
	Untreated	10	N/D	N/D	N/D	N/D	N/D	N/D

Treated water includes drinking water, water dispenser, mineral water, tap water and swimming pools

Untreated water includes rain water, springs, wells, recreational lake, rivers, waterfalls, canals/channels and effluent water

^a^N/D means not detected

#### Untreated water

Table 2 shows the presence of *Cryptosporidium* oocysts in untreated water samples from three countries, namely the Philippines (47.8 %), Malaysia (38.6 %), and Thailand (26.9 %). The highest oocyst concentration was recorded in water samples from Malaysia, with a concentration of 0.57 ± 1.41 oocyst/L, followed by samples from Thailand (0.22 ± 0.59 oocyst/L) and the Philippines (0.13 ± 0.18 oocyst/L).

Using the qPCR method, *C. parvum* was detected in 4.5 % of the samples from Malaysia (Ct: 23.97 ± 3.8; CV: 0.16) and 4.3 % of the samples from the Philippines (Ct: 36.53), as summarized in Table 4.

A high presence of oocysts was detected in untreated water samples compared to treated water from the aforementioned countries. *C. parvum* was found only in untreated water samples from Malaysia and the Philippines.

### Detection of *Giardia* spp.

#### Treated water

*Giardia* cysts were detected in one treated water sample from the Philippines (10 %), out of 10 total samples, with a concentration of 0.02 ± 0.06 cyst/L (see Table 2). Only one sample showed the presence of *G. lamblia* using the qPCR method (10 %), with a Ct value of 32.26 ± 0 (see Table 4).

#### Untreated water

*Giardia* cysts were detected in untreated water samples from the Philippines (8.90 ± 19.65 cyst/L), Malaysia (0.92 ± 1.74 cyst/L), Vietnam (0.51 ± 0.81 cyst/L), and Thailand (0.12 ± 0.30 cyst/L), as shown in Table 2. The Philippines had the highest number of samples positive for *G. lamblia* (73.9 %)*,* followed by Malaysia (27.3 %) and Thailand (22.6 %), with Ct values ranging from 30.49 to 34.41 and CVs ranging from 0.1 to 0.18 (see Table 4). Thus, it can be deducted that *Giardia* cysts and *G. lamblia* are frequently detected in untreated water.

### Positive correlations between (oo)cyst concentration and physicochemical parameters

Table 5 shows correlation values between the concentrations of various physicochemical parameters and (oo)cysts.

**Table 5:** Correlation between (oo)cysts concentration and physicochemical parameters

Country	Type of water	Correlation of (oo)cyst concentration and physicochemical parameter(s)										
			Oocyst	Cyst	Chlorine	Ammonia	Nitrate	Nitrite	DO	TDS	Salinity	Turbidity
Malaysia	Treated	Oocyst	1.000									
		Cyst	0	1.000								
		Chlorine	0	0	1.000							
		Ammonia	0	0	−0.183	1.000						
		Nitrate	0	0	0.182	−0.261	1.000					
		Nitrite	0	0	−0.171	−0.155	−0.284	1.000				
		DO	0	0	0.224	−0.305	0.303	−0.367	1.000			
		TDS	0	0	−0.010	−0.196	−0.088	−0.111	0.027	1.000		
		Salinity	0	0	−0.344	0.181	−0.433	−0.113	−0.488	0.733	1.000	
		Turbidity	0	0	−0.181	−0.232	0.237	−0.191	0.846	−0.255	−0.583	1.000
	Untreated	Oocyst	1.000									
		Cyst	0.590	1.000								
		Chlorine	−0.031	−0.099	1.000							
		Ammonia	0.421	0.742	0.181	1.000						
		Nitrate	0.441	0.603	0.082	0.434	1.000					
		Nitrite	0.473	0.614	0.172	0.597	0.851	1.000				
		DO	−0.022	−0.078	−0.307	−0.317	−0.126	−0.326	1.000			
		TDS	0.156	0.284	0.221	0.327	−0.076	−0.042	0.217	1.000		
		Salinity	0.270	0.458	0.448	0.657	0.133	0.337	−0.388	0.718	1.000	
		Turbidity	−0.043	−0.081	0.228	−0.005	0.040	−0.027	−0.292	0.226	0.240	1.000
Thailand	Treated	Oocyst	1.000									
		Cyst	0	1.000								
		Chlorine	0	0	1.000							
		Ammonia	0	0	−0.221	1.000						
		Nitrate	0	0	0.735	0.037	1.000					
		Nitrite	0	0	0.518	0.444	0.772	1.000				
		DO	0	0	−0.088	−0.244	−0.118	0.085	1.000			
		TDS	0	0	−0.027	0.236	0.639	0.550	−0.087	1.000		
		Salinity	0	0	−0.044	0.259	0.627	0.535	−0.103	0.998	1.000	
		Turbidity	0	0	−0.008	0.306	−0.233	0.173	−0.077	−0.200	−0.215	1.000
	Untreated	Oocyst	1.000									
		Cyst	0.838	1.000								
		Chlorine	0.037	0.117	1.000							
		Ammonia	0.153	0.179	0.120	1.000						
		Nitrate	−0.034	0.031	0.423	0.299	1.000					
		Nitrite	0.137	0.169	0.404	0.446	0.657	1.000				
		DO	−0.072	−0.035	−0.124	−0.242	−0.037	−0.148	1.000			
		TDS	−0.147	−0.176	0.262	−0.252	0.090	0.162	0.200	1.000		
		Salinity	0.205	0.215	−0.185	0.135	−0.295	−0.180	0.410	−0.166	1.000	
		Turbidity	0.251	0.334	0.207	0.347	0.067	0.232	0.171	−0.096	0.517	1.000
Philippines	Treated	Oocyst	1.000									
		Cyst	1.000	1.000								
		Chlorine	−0.339	−0.339	1.000							
		Ammonia	−0.155	−0.155	−0.259	1.000						
		Nitrate	0.993	0.993	−0.260	−0.178	1.000					
		Nitrite	0.272	0.272	−0.146	−0.414	0.199	1.000				
		DO	−0.445	−0.445	0.164	−0.098	−0.407	−0.463	1.000			
		TDS	0.447	0.447	0.342	−0.033	0.436	0.481	−0.532	1.000		
		Salinity	0.447	0.447	0.338	−0.048	0.435	0.497	−0.539	1.000	1.000	
		Turbidity	−0.359	−0.359	−0.251	−0.452	−0.364	0.011	0.388	−0.827	−0.816	1.000
	Untreated	Oocyst	1.000									
		Cyst	0.516	1.000								
		Chlorine	0.310	−0.113	1.000							
		Ammonia	0.297	0.450	0.330	1.000						
		Nitrate	0.191	0.325	0.570	0.500	1.000					
		Nitrite	0.107	0.720	−0.020	0.614	0.578	1.000				
		DO	−0.354	0.084	−0.879	−0.507	−0.533	−0.016	1.000			
		TDS	0.188	−0.134	0.484	−0.042	0.547	−0.128	−0.408	1.000		
		Salinity	0.151	−0.159	0.527	0.121	0.059	−0.057	−0.470	−0.105	1.000	
		Turbidity	0.337	0.127	0.600	0.690	0.288	0.146	−0.596	0.138	0.252	1.000
Vietnam	Treated	Oocyst	1.000									
		Cyst	0	1.000								
		Chlorine	0	0	1.000							
		Ammonia	0	0	−0.939	1.000						
		Nitrate	0	0	−0.302	0.187	1.000					
		Nitrite	0	0	1.000	−0.937	−0.312	1.000				
		DO	0	0	−0.361	0.642	0.141	−0.360	1.000			
		TDS	0	0	−0.353	0.101	−0.305	−0.349	−0.661	1.000		
		Salinity	0	0	−0.287	−0.022	−0.086	−0.286	−0.779	0.960	1.000	
		Turbidity	0	0	0.996	−0.952	−0.357	0.996	−0.428	−0.266	−0.207	1.000
	Untreated	Oocyst	1.000									
		Cyst	0	1.000								
		Chlorine	0	0.084	1.000							
		Ammonia	0	0.648	0.807	1.000						
		Nitrate	0	0.181	0.965	0.847	1.000					
		Nitrite	0	0.500	0.782	0.876	0.882	1.000				
		DO	0	−0.628	0.274	−0.176	0.031	−0.332	1.000			
		TDS	0	0.531	0.761	0.879	0.867	0.999	−0.365	1.000		
		Salinity	0	0.531	0.760	0.879	0.867	0.999	−0.367	1.000	1.000	
		Turbidity	0	−0.018	0.978	0.730	0.971	0.801	0.219	0.778	0.778	1.000

Treated water includes drinking water, water dispenser, mineral water, tap water and swimming pools

Untreated water includes rain water, springs, wells, recreational lake, rivers, waterfalls, canals/channels and effluent water

In Malaysia, TDS detected in treated water samples had a positive correlation with salinity (0.733). Meanwhile, in untreated water samples, salinity had a positive correlation with ammonia (0.657) and TDS (0.708), whereas nitrite also had a high positive correlation with nitrate (0.851). Amongst the parameters, ammonia, nitrate, and nitrite (0.603–0.742) each had a positive correlation with *Giardia* cyst concentrations in untreated water samples.

In Thailand, the data from treated water samples revealed positive correlations between nitrate and salinity (0.627), nitrate and TDS (0.639), nitrate and nitrite (0.772), and nitrate and chlorine (0.735). Nitrite had a positive correlation with chlorine (0.518), salinity (0.535), TDS (0.55), while salinity showed a positive correlation with TDS (0.998). In untreated water samples, a positive correlation was observed between turbidity and salinity (0.577), and nitrite and nitrate (0.656).

In the Philippines, a positive correlation was observed between nitrate and (oo)cyst concentration (0.993), salinity and (oo)cyst concentration (1), and TDS and (oo)cyst concentration (1) in treated water samples. Positive correlations were also observed between (oo)cyst and cyst concentrations (0.516), salinity and chlorine (0.527), nitrate and TDS (0.547), turbidity and chlorine (0.6), ammonia and nitrite (0.614), ammonia and turbidity (0.69), and nitrite and cyst concentration (0.720) in untreated water samples.

In Vietnam, there was a positive correlation between DO and ammonia (0.642), salinity and TDS (0.96), chlorine and turbidity (0.996), and chlorine and nitrite (1) in the treated water samples. In untreated water samples, ammonia was positively correlated with cyst concentration and turbidity, with values of 0.648 and 0.73, respectively. There was a positive correlation between turbidity and both TDS and salinity (0.778), whereas ammonia had a positive correlation with nitrate, nitrite, and both TDS and salinity, with values of 0.847, 0.876, and 0.879, respectively. Nitrate also showed positive correlation with nitrite (0.882), both TDS and salinity (0.867), and turbidity (0.971), whereas salinity and TDS were both positively correlated with nitrite (0.999).

Overall, positive correlations with the presence of parasites were obtained from the chemicals parameters of ammonia, nitrate, and nitrite from Malaysia, the Philippines, and Vietnam.

## Discussion

In recent years, the SEA region has seen an exponential increase in the number of residents, with an estimated total population of 290.5 million in the four studied countries. The growing population has meant that there is an urgent need for clean water for daily usage, however, the ability to provide a potable water supply is rather limited in some countries due to the costs involved in building dams. Slow economic growth in some countries in SEA has been a limiting factor in preparing, treating, and supplying clean water to the inhabitants. Clean and treated water is readily available in urban areas but in rural areas, people still depend on natural water sources, such as rainwater, rivers, lakes, etc., and these may have become contaminated by waterborne protozoan parasites.

Physicochemical data can be used to preliminary determine water quality, and play a vital role in reflecting nutrient availability which enables parasites to survive. For example, fluctuations in TDS can affect the pH level and DO of the water both directly and indirectly, depending on particle matters. The increase in TDS, and DO are mainly due to increased water usage and precipitation, and are unfavorable conditions for the survival of (oo)cysts, as shown by the negative correlations between (oo)cysts and these parameters in the present study. Previous studies have postulated to use the correlation of nitrate and nitrite concentrations to predict the presence of (oo)cysts in water [20]. Moreover, the present study revealed that ammonia had a positive correlation with oocysts, hence suggesting the need for further evaluate how the aforementioned parameters can be used as indicators of the presence of (oo)cysts in both treated and untreated water samples.

This study showed the existence of an interesting physicochemical relationship between the presence of *Cryptosporidium* and *Giardia*. It has been proven that turbidity is positively correlated with a number of (oo)cysts due to run-off intensity and effluent discharge. However, in this study, a negative correlation between (oo)cysts and turbidity was observed. Supporting this, another study has demonstrated that the recovery rate of *Cryptosporidium* oocysts and *Giardia* cysts declined as the turbidity level in the water increased, regardless of the filtration method used [23, 24].

Mostly it was the untreated water samples in this study that were contaminated with parasites. The presence of protozoan parasites in waterfalls could explain how humans become directly exposed to parasitic infections (through recreational activities such as bathing and swimming in such waterfalls). Water basins have also been polluted due to high water usage for domestic, fishing, and recreational purposes by local inhabitants living near rivers. Fortunately, there were no *Cryptosporidium* oocysts and *Giardia* cysts found in the selected treated water samples from Malaysia, Thailand, and Vietnam. This means that the process of treating water can effectively eliminate (oo)cysts.

In this study, *Giardia* cysts (cyst/L) appeared to be a major contaminant, and were detected in untreated water samples at a rate of 11.4 times higher than *Cryptosporidium* oocysts (oocysts/L). This finding is confirmed by the common discovery of *Giardia* cysts in raw water [25]. This is possibly due to the size of the *Giardia* cysts (10 to 15 μm in length; 7 to 10 μm in width) and its thickness (0.3 to 0.5 μm), compared to *Cryptosporidium* oocysts, as reported by the EPA [26]. Due to their relatively bigger size, these cysts can be easily trapped on filter paper.

In this study, we chose to purify recovered (oo)cysts using filtration and concentrated them via centrifugation without using any chemicals that are potential PCR inhibitors [27, 28]. These inhibitors can affect the amplification by inactivating the thermostable DNA polymerase and/or by interfering with nucleic acids [29, 30]. Morphological identification was performed through microscopy and molecular screening was conducted using the qPCR method, independently, to obtain a useful and valid comparison between the methods.

Although it has been widely proven that the IMS purification technique is considered to be an efficient step pre-PCR, the presence of foreign particles is inevitable (humus, sediment, etc.) due to poor water quality (cloudy and turbid) [31]. Disturbances of the fluorescent signal generated by bead opacity should be avoided after observation. Both these scenarios will cause interference in the fluorescent signal during PCR assay and lead to inhibiting effects, which are known to decrease PCR efficiency. Thus, more purification steps are necessary for the separation and isolation of (oo)cysts from the slides, which can permit lower (oo)cysts recoveries and DNA yield, prior to molecular analysis. Since there is a low number of *Cryptosporidium* oocysts and *Giardia* cysts in environmental samples, even a small loss can have a significant influence on their detection via quantitative PCR [32].

Not knowing the time when the (oo)cysts were released into water bodies, the extended time needed for processing samples in the lab until molecular analysis can begin, and the loss that occurs during the physical lysis technique and centrifugation steps might contribute to low recoveries during molecular analysis. These factors could also explain the low level or absence of intact (oo)cysts that are needed to maintain good DNA integral contents [33]. Furthermore, viable (oo)cysts (DNA identification via DAPI staining) were not quantified in this study. Thus, (oo)cysts viably observed by microscopy do not determine the exact quantity confirmed by the qPCR method. In fact, the loss of intact DNA may be due to uncertain conditions such as aged (oo)cysts that were recovered from the samples, distorted (oo)cysts, or (oo)cysts that were removed by the flow of water during sampling, making the concentration of the DNA template below the detection limit for PCR assay.

The IMS technique has been the subject of many studies globally and is considered the gold standard for the identification of (oo)cysts [34]. However, it is also time-consuming, laborious, and tedious. Moreover, it has a major drawback: cross-reaction with non-target organisms (algae, debris, etc.) and with cysts of other protozoan parasites, in addition to contributing to the loss of (oo)cysts during isolation and purification. The IMS method is also unable to provide specific identification for *C. parvum* and *G. lamblia* due to a lack of antibody specificity.

Real-time PCR can provide comparable data to that acquired using the IMS technique without compromising the number of samples. The selection of primer-probe targets is based on the unique molecules of each organism, the biology of which are equally important. Detection of these parasites at a molecular level also takes into account the sensitivity and specificity of each primer-probe used in the PCR method [35, 36]. It is crucial to determine the effectiveness of the analysis in order to avoid cross-reactivity among species. The primer-probe specificity for molecular analysis can be tested beforehand for a wide range of protozoan parasites, so that only targeted amplicons are generated prior to sequencing and the DNA sequence of interest can be represented. Although there used to be many types of PCR, such as nested PCR and RFLP sequencing, currently there is a lack of studies published on parasitic DNA detected in water samples of 10 l and above. However, the sensitivity of qPCR is high and able to detect even trace amounts of DNA in an (oo)cyst.

Some environmental factors can also be used to predict the incidence of (oo)cysts in water bodies. For example, meteorological data (i.e. on rainfall) can affect the leachate sediment process after downpour, thus influencing the turbidity and TDS levels of water bodies. Moderate to high correlations were observed between the eight physicochemical parameters employed in this study. Other studies have also pointed to significant interactions of waterborne parasites with other chemical and biological factors [37, 38].

## Conclusion

Based on the results obtained, physicochemical parameters can be used to obtain baseline data on the deterioration of water quality and how this is correlated with parasite occurrence (i.e. (oo)cyst). Real-time PCR assay allows for feasible detection and identification of *C. parvum* oocysts and *G. lamblia* cysts purified from various types of water samples. The qPCR method can be used for large samples, and is useful for understanding the abundance and distribution of protozoan parasites across different geographic locations. The presence of *C. parvum* and *G. lamblia* in both raw and treated water samples can result in potential risk for zoonotic disease transmission in SEA. This requires the urgent attention of relevant policymakers who can implement the necessary preventive measures.

## Abbreviations

CI: confidence interval
Ct: threshold cycle
CV: coefficient of variation
DO: dissolved oxygen
EPA: The US Environmental Protection Agency
IMS: immunomagnetic separation
PCR: polymerase chain reaction
PCR-RFLP: polymerase chain reaction-restriction fragment length polymorphism
qPCR: real-time polymerase chain reaction
SD: standard deviation
SEA: Southeast Asia
TDS: total dissolved solids
